# Evaluation of the Trueness and Precision of a Simplified Method for Transferring the Relationship of the Maxillary Plane and Transverse Hinge Axis to Virtual Articulators

**DOI:** 10.1155/ijod/2778184

**Published:** 2026-06-05

**Authors:** Chaimongkon Peampring, Leena Kiratiwudh

**Affiliations:** ^1^ Department of Prosthetic Dentistry, Faculty of Dentistry, Prince of Songkla University, Hat Yai, 90110, Thailand, psu.ac.th; ^2^ Galyanivadhanakarun Hospital, Princess of Naradhiwas University, Khok Khian, Mueang Narathiwat District, Narathiwat, 96000, Thailand, pnu.ac.th

**Keywords:** accuracy, condylar path, occlusal plane, virtual articulator

## Abstract

**Background:**

The digital transfer of data for establishing the maxillary occlusal plane and condylar path aims for high precision, facilitating accurate use of virtual articulators and aiding in digital prosthetic fabrication.

**Objective:**

This study evaluates the accuracy of transferring maxillary scans to a virtual articulator using a novel customized transfer tool compared to a conventional facebow system.

**Methods:**

Three types of master models, bilateral balanced, monoplane, and lingualized occlusion, were prepared and duplicated. Landmarks on the maxillary and mandibular models were identified, and interlandmark distances served as controls. The models were scanned intraorally, and virtual bite alignment was performed using a best‐fit algorithm. A manual facebow transfer defined the occlusal and condylar relationships on a mechanical articulator, which was then transferred to a virtual articulator via the customized transfer tool and computer‐aided design (CAD) software. Transfer accuracy was assessed by comparing interlandmark distances between mechanical and virtual setups using statistical tests (Shapiro–Wilk, Levene’s, and *t*‐tests) at *α* = 0.05. Reliability was evaluated with the intraclass correlation coefficient (ICC).

**Results:**

No significant differences were found between the mechanical and virtual interlandmark distances across all models (*p* > 0.05). Differences ranged from −0.08 to 0.11 mm, within clinically acceptable limits. ICC values exceeded 0.80, indicating high reliability of the transfer method.

**Conclusions:**

The customized transfer tool accurately transferred maxillary occlusal plane and condylar path relationships to the virtual articulator, with no significant discrepancies observed.

## 1. Introduction

An articulator is an essential instrument used in laboratory procedures, playing a crucial role in both diagnostics and treatment planning for oral reconstruction. While mechanical articulators will continue to hold importance, virtual articulators are gaining traction in improving clinical outcomes, particularly with the rise of computer‐aided design and manufacturing (CAD/CAM) technologies [1, 2]. In prosthodontic rehabilitation, accurately positioning dental models within an articulator is vital for achieving a functional and precise occlusion and for transferring the correct relationship of the upper occlusal plane to the condylar path [3]. The ultimate fit and clinical quality of a prosthesis are inherently linked to how accurately dental models are oriented within the articulator [4, 5].

Consideration of the virtual articulator system utilized in CAD software is crucial when determining the appropriate reference for the maxillary occlusal plane [2]. This is vital to ensure that the occlusal design of the CAD/CAM prosthesis can be accurately validated through the simulation of dynamic movements, including protrusive and excursive motions, on the virtual articulator [1, 2]. Therefore, the precise transfer of digital data to the virtual articulator, in alignment with the patient’s anatomical reference points pertaining to the maxillary occlusal plane and condylar pathway, is essential for the effective simulation of mandibular movement within the CAD environment [5, 6]. Digital positioning of data onto a virtual articulator can be accomplished through advanced techniques such as 3D facial scanning [7] and full‐face cone‐beam computed tomography (CBCT) imaging [6, 8]. Nonetheless, the substantial costs associated with these sophisticated imaging modalities may hinder their widespread implementation in clinical practice. Consequently, this study aims to propose an alternative methodology for transferring the relationship between the maxillary occlusal plane and the condylar path to the virtual articulator by employing a customized transfer tool in conjunction with the conventional physical facebow utilized in everyday practice. Additionally, the accuracy of this customized transfer tool, referred to as “SmartBox,” was evaluated by comparing interlandmark distances from reference points to assess the differences in articulation between mechanical articulators and virtual articulators.

## 2. Materials and Methods

This study was granted an exemption by the Ethics Committee of the Faculty of Dentistry, Prince of Songkla University (PSU.109.1.6.3/67‐0211).

**Figure 1:**
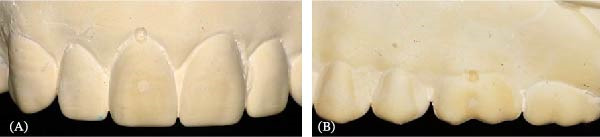
(A) Anterior landmark on tooth 11 and (B) posterior landmark on tooth 26.

Dentate maxillary and mandibular models were constructed using acrylic artificial teeth (Majordent) to represent three patients with differing occlusal patterns as reference standards. The first model displayed maxillary and mandibular teeth arranged in a bilateral balanced occlusion, employing anatomical teeth for both the maxillary and mandibular arches. The second model was designed with teeth arranged in a monoplane occlusion, utilizing nonanatomic teeth in both arches. The third model incorporated a lingualized occlusion, featuring anatomical teeth in the maxillary arch and semi‐anatomic teeth in the mandibular arch. These arrangements allowed for guidance solely by the posterior determinants of the articulator and the incisal table, ensuring that no interference arose from the cusps of the mandibular teeth during dynamic movements of the articulator. Each of the arranged tooth models was duplicated using irreversible hydrocolloid impressions (Kromopan) and poured with type‐IV dental stone (Kromotypo 4) to produce three sets of master models. To establish reproducible reference points, 1‐mm‐deep fiducial indentations were milled into the master models using a pointed carbide bur. This depth was chosen to accommodate the measuring tips of a digital vernier caliper. The indentations were made at the gingival margin of the mid‐buccal surfaces of the first molars in both arches, as well as at the right upper and lower incisors of each model. These landmarks acted as reference points for measuring interlandmark distances (Figure 1).

**Figure 2:**
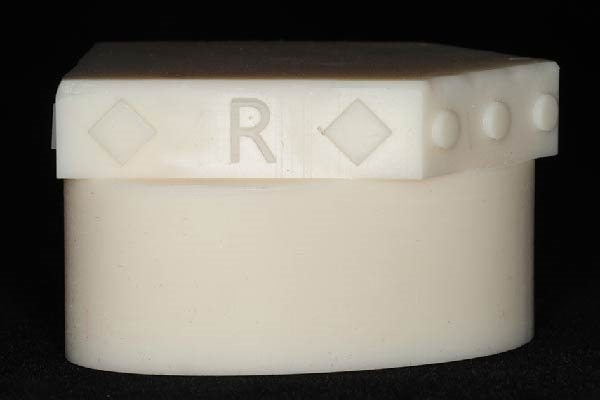
The customized transfer tool (SmartBox) with landmarks for superimposition.

Each matched pair of master models was articulated in a semi‐adjustable articulator (Pro Arch Articulator IIIEG) using a simulated arbitrary facebow (Pro Arch Face Bow) in conjunction with a bite fork. The models were positioned in maximum intercuspation, with the incisal guide table and condylar guidance adjusted to angles of 10° and 30°, respectively. Interlandmark distances were measured from the established reference points using a digital vernier caliper with an accuracy of ±0.001 inch (Point Caliper 573‐721‐20). Measurements were taken for both maximum intercuspation and protrusive movement of the master models (Figure 2). During the protrusive movement, the models were guided until the incisal edges of the maxillary and mandibular central incisors were aligned at the midline. A protrusive record was subsequently captured at the maxillary and mandibular incisors using pattern resin (GC Pattern Resin) to ensure consistency in repeated measurements. To minimize operator error, each distance was measured 30 times, and statistical analysis was performed on the collected data. The mean values obtained from the mechanical articulators served as the standard reference values for comparison.

The maxillary and mandibular master models from each group were digitized using an intraoral scanner (TRIOS 3). Interocclusal registration was performed through a bilateral buccal scan, conducted 10 times for each model to simulate 10 samples per group. The sample size (*n* = 10 per group) was determined based on a power analysis (G
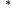
Power v3.1.9.7) using data from a pilot study. To detect a large effect size (*f* = 0.80) with an alpha level of 0.05 and a power of 0.80, the analysis indicated that a minimum sample size of 9 per group was required. Therefore, a sample size of 10 was selected to ensure adequate statistical power. This sample size is consistent with previously published in vitro validation studies evaluating the accuracy of virtual articulators and digital transfer methods. The maxillary and mandibular models were virtually aligned with the digital interocclusal records utilizing a best‐fit algorithm, ensuring precise alignment without manual intervention.

**Figure 3:**
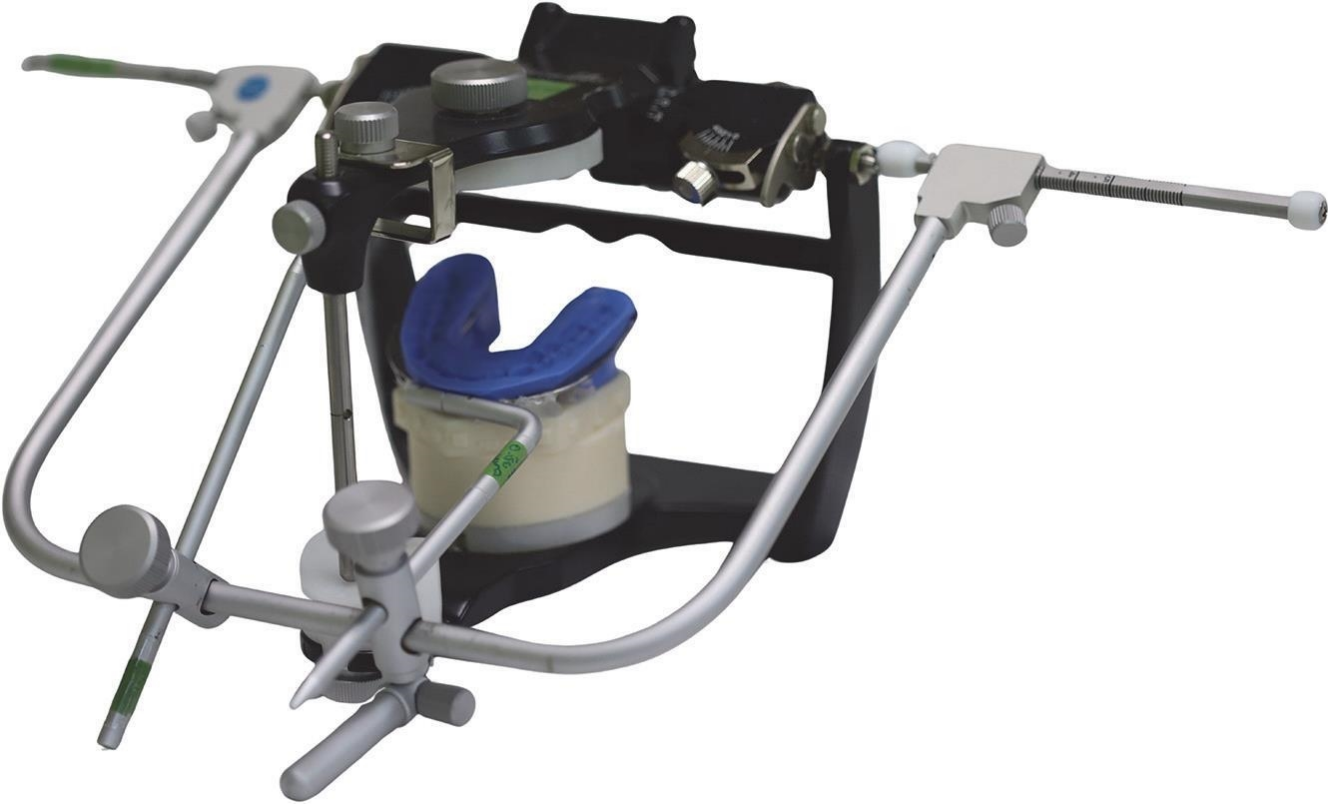
The customized transfer tool positioned in mechanical articulator and arbitrary facebow.

**Figure 4:**
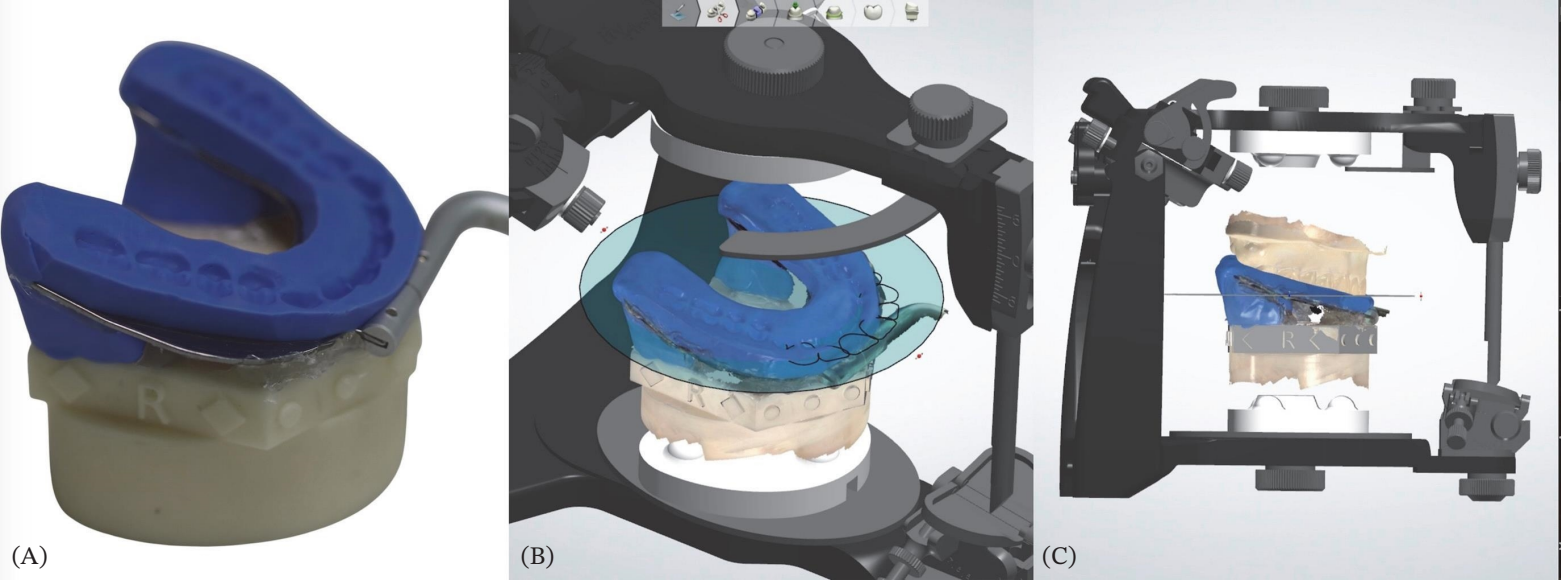
(A) Indentation of maxillary occlusal surface in relation to SmartBox, (B) superimposition of scanned SmartBox to the virtual SmartBox in CAD software, and (C) maxillary scan model was aligned to match the indentation of maxillary occlusal surface on the bite fork, completing the transfer process.

**Figure 5:**
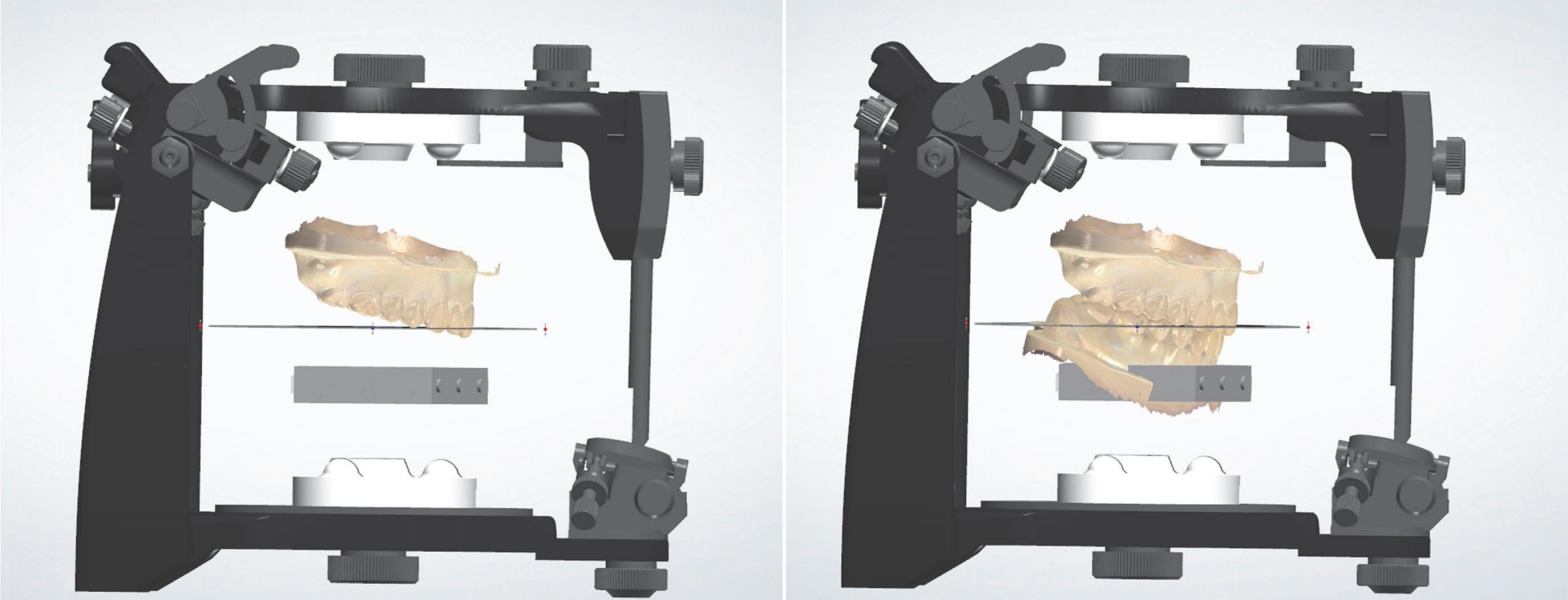
The bite fork was concealed, and the mandibular scan model was aligned with the maxillary scan model in the maximum intercuspal position.

**Figure 6:**
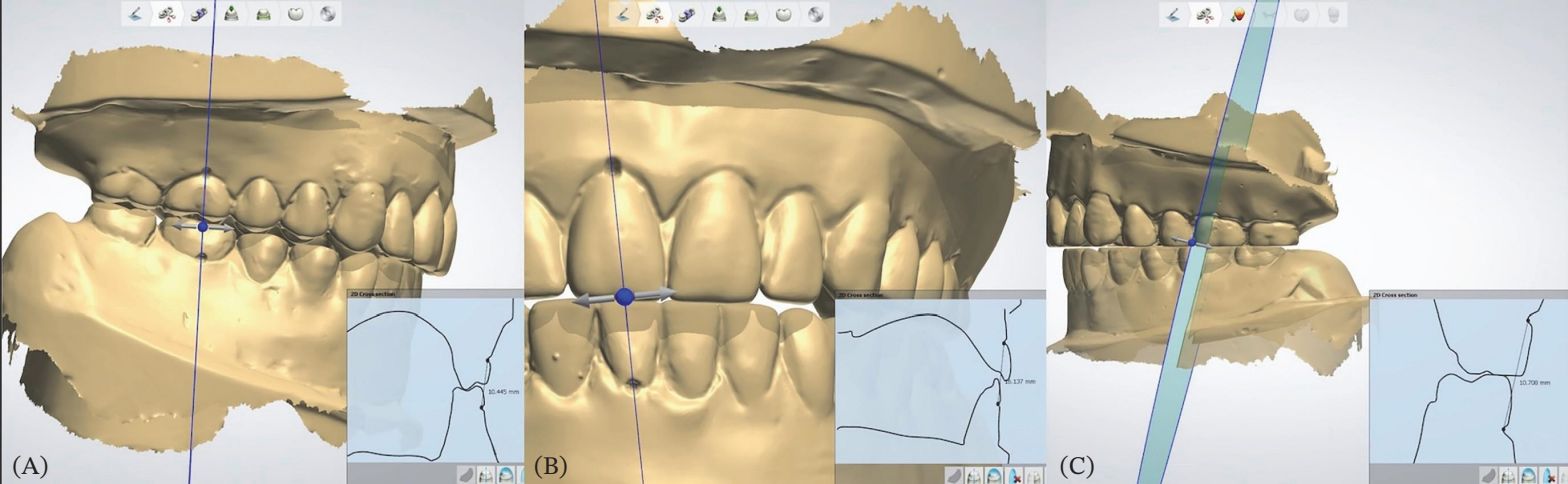
Measurement of interlandmark distances from reference points in each model using the 2D cross‐section tool. (A) Right posterior, (B) anterior, and (C) left posterior landmarks.

**Figure 7:**
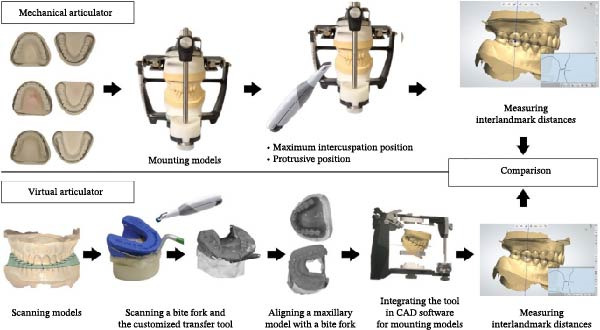
Summary of comparison between mechanical and virtual workflows.

To accurately establish the relationship between the maxillary occlusal plane and the condylar path of the mounted models for subsequent transfer to a virtual articulator, a facebow transfer was executed using an arbitrary facebow (Pro Arch Face Bow). The bite fork was filled with putty‐type polyvinyl siloxane (PVS) (Honigum Putty) and securely positioned onto the maxillary reference model to capture the impression of the maxillary occlusal surfaces, with particular attention paid to aligning the midline of the bite fork with the articulator’s midline. Subsequently, the facebow arm was affixed to the mechanical articulator (Pro Arch Articulator IIIEG) by aligning the ear piece of the facebow with the ear location pin of the articulator. Once the relationship between the maxillary occlusal plane and the condylar path was successfully recorded, both the maxillary and mandibular models were detached from the articulator, while the facebow and registered bite fork remained in place. To facilitate the transfer of the established relationship to the virtual articulator, the customized transfer tool, termed the SmartBox, was positioned on the lower member of a mechanical articulator. SmartBox was digitally designed using CAD software (Autodesk Meshmixer). The tool features a cylindrical geometry with a height of 25 mm and a radius of 20 mm. To facilitate the digital alignment (merging) process, the lateral surface of the cylinder incorporates specialized geometric landmarks, including rectangular extrusions and alphanumeric identifiers (Figure 3). These features provide unique topographical data points for the “best‐fit” algorithm during the superimposition of the intraoral scan with the CAD library file. The digital transfer workflow began with capturing the maxillary relationship using a mechanical facebow (Pro Arch Face Bow) and a PVS bite fork record. The SmartBox was then securely positioned on the lower member of the mechanical articulator, and an intraoral scanner (TRIOS 3) was used to scan the SmartBox in direct relation to the registered bite fork while it was still attached to the facebow assembly (Figure 4). The resulting STL file was imported into the CAD software (3Shape Dental System), where the scanned SmartBox was superimposed onto the corresponding virtual SmartBox within the CAD library using the unique rectangular and alphanumeric landmarks. This alignment process automatically oriented the maxillary scan to the virtual articulator’s hinge axis, effectively replicating the mechanical mounting in a digital environment (Figure 5). All models were mounted to the virtual articulator using an identical method. The incisal guide table and condylar guidance of the virtual articulator were set to 10° and 30°, respectively (Figure 6). To assess the accuracy of the transfer process, interlandmark distances were measured from reference points in each model using the 2D cross‐section tool in the 3Shape Dental System software (Figure 7). These measurements were compared to the interlandmark distances obtained manually from the mechanical articulator. A total of 10 scans from the virtual articulator group were collected for statistical analysis.

Data from interlandmark distance measurements at the right posterior, anterior, and left posterior reference points of each model in both maximum intercuspation and protrusive movement positions were analyzed for normal distribution using the Shapiro–Wilk test and for homogeneity of variance using Levene’s test. A one‐sample *t*‐test was employed to compare the means, assessing significant differences between the mechanical articulator and virtual articulator groups, with the significance level set at *α* = 0.05. The summary of the comparison between mechanical (considered the standard) and virtual workflows is illustrated in Figure 7. Additionally, the intraclass correlation coefficient (ICC) was calculated to assess the reliability of transferring interlandmark distances from the mechanical articulator to the virtual articulator.

## 3. Results

All recorded data from the mechanical articulator and virtual articulator conformed to the assumptions of normality and homogeneity of variance, with no significant differences observed between the two groups. Comparisons of measurements were conducted for three anatomical landmarks (right posterior, anterior, and left posterior) across all models in both the maximum intercuspation position and the protrusive position. The results indicated that the mean interlandmark distance ± standard deviation values for the mechanical articulator and virtual articulator were consistent, as presented in Tables 1 and 2. Furthermore, the ICC results are presented in Table 3. The results indicated that the tool provided high reliability in transferring this relationship, with ICC values exceeding 0.8 at all reference points, which is indicative of good reliability [9].

**Table 1:** Mean interlandmark distance ± standard deviation (mm) in manual articulator versus virtual articulator in maximum intercuspation position.

Model	Position	Manual articulator	Virtual articulator	*P* (*α* = 0.05)
1	Rt	11.03 ± 0.10	11.01 ± 0.06	0.290
	Ant	16.14 ± 0.23	16.15 ± 0.15	0.867
	Lt	11.05 ± 0.22	11.07 ± 0.16	0.734

2	Rt	10.69 ± 0.17	10.68 ± 0.12	0.883
	Ant	17.10 ± 0.18	17.00 ± 0.19	0.134
	Lt	10.80 ± 0.21	10.81 ± 0.16	0.918

3	Rt	10.32 ± 0.53	10.34 ± 0.53	0.906
	Ant	16.14 ± 0.08	16.13 ± 0.10	0.798
	Lt	10.24 ± 1.01	10.13 ± 0.53	0.533

**Table 2:** Mean interlandmark distance ± standard deviation (mm) in manual articulator versus virtual articulator in protrusive position.

Model	Position	MA standard value	VA	*P* (*α* = 0.05)
1	Rt	12.34 ± 0.21	12.42 ± 0.16	0.145
	Ant	17.10 ± 0.21	17.08 ± 0.21	0.789
	Lt	12.54 ± 0.78	12.59 ± 0.53	0.806

2	Rt	11.12 ± 0.93	11.20 ± 0.53	0.627
	Ant	16.71 ± 0.22	16.61 ± 0.21	0.139
	Lt	11.73 ± 0.19	11.72 ± 0.19	0.883

3	Rt	11.32 ± 0.17	11.25 ± 0.12	0.109
	Ant	16.85 ± 0.22	16.87 ± 0.22	0.777
	Lt	11.26 ± 0.84	11.30 ± 0.53	0.795

**Table 3:** Intraclass correlation coefficient between manual articulator and virtual articulator.

Model	Maximum intercuspation position			Protrusive position		
	Right	Anterior	Left	Right	Anterior	Left
1	0.91	0.83	0.86	0.86	0.94	0.91
2	0.81	0.89	0.91	0.83	0.93	0.86
3	0.92	0.83	0.89	0.91	0.81	0.85

## 4. Discussion

Establishing a precise maxillomandibular orientation is the cornerstone of successful complex prosthetic reconstruction. Utilizing a physical facebow allows clinicians to capture the spatial relationship of the dentition relative to the hinge axis, providing the necessary foundation for analyzing mandibular kinematics and planning restorative outcomes [10]. Recent advancements in digital dentistry are transitioning articulators from mechanical to virtual formats. Proper transfer of digital data to a virtual articulator requires alignment with the patient’s anatomical reference plane, making the maxillary occlusal plane selection critical to the articulator system used in CAD software [1, 2, 11–13].

The integration of the maxillary occlusal plane into a virtual environment has been previously explored through several sophisticated methodologies. While several digital strategies emphasize the fusion of CBCT data with 3D facial scans to orient the maxillary arch [14], these sophisticated modalities often involve a steep financial threshold and operational complexities that preclude their use in everyday clinical settings [6–8, 15].

Other digital techniques utilize specialized alignment jigs or 2‐piece digital facebow systems designed to bridge intraoral scans with virtual articulators [16]. Our proposed SmartBox method differs from these approaches by offering a hybrid workflow. Instead of bypassing traditional equipment, it utilizes the existing mechanical facebow, a tool already familiar to clinicians, and converts that physical relationship into a digital format via a specific transfer tool.

This study proposes a customized transfer tool (SmartBox) to enhance the accuracy of the maxillary occlusal plane and condylar path transfer to a virtual articulator, integrating a mechanical articulator into the digital workflow. The primary advantage of the SmartBox is its ability to maintain the accuracy of the mechanical “arbitrary facebow” transfer while eliminating the need for complex digital hardware like electronic facebows or facial scanners. This makes the transition to a virtual articulator more accessible and cost‐effective, particularly in settings where traditional facebows are already part of the standard clinical protocol. By utilizing multiple referent landmarks for superimposition, the SmartBox minimizes the alignment errors often seen in manual “best‐fit” alignments in CAD software, providing a standardized and repeatable bridge between the physical and virtual worlds. This study aimed to assess the accuracy of this transfer process, with previous literature reporting successful static occlusal analyses, despite a scarcity of studies focused on dynamic jaw movements [17, 18].

While static position analyses have proven useful, further research into eccentric positions could enhance the understanding of jaw relationships, ultimately informing treatment protocols that promote ideal occlusion and stability for patients [7]. To validate the transfer protocol, we employed linear interlandmark assessments across three key reference zones: the bilateral posterior regions and the anterior midline. These specific metrics were selected because of their high degree of reproducibility and their sensitivity in detecting minor spatial deviations during both static and eccentric mandibular positions.

Statistical analysis revealed no significant differences between interlandmark distances of mechanical articulator and virtual articulator measurements, confirming successful transfer accuracy with *p*‐values exceeding 0.05 across all comparisons. The mean differences ranged from −0.08379 to 0.1083 mm, consistent with the literature indicating minimal clinical significance in deviations. Therefore, the null hypothesis was fully accepted [19]. The ICC values were used to assess the reliability of SmartBox. Results indicated that the tool provided high reliability in transferring this relationship from the mechanical articulator to the virtual articulator, with ICC values exceeding 0.8 at all reference points [9]. To validate the spatial transfer of the maxillary arch beyond simple static alignment, this study utilized protrusive movement measurements as a proxy for orientation accuracy. Because the path of any point on the mandible is determined by its starting position relative to the rotation centers (transverse hinge axis), a linear measurement in an eccentric position effectively captures potential errors in the 3D orientation. If the virtual mounting was spatially inaccurate, the arc of movement would deviate, and the linear distances between landmarks during protrusion would not match the mechanical standard. The high level of agreement and high ICC values found in our protrusive data suggest that the SmartBox method successfully replicates the spatial relationship between the maxillary plane and the transverse hinge axis.

Despite the promising results, the current in vitro design inherently lacks the biological variables present in a clinical environment. Additionally, it is important to note that the fidelity of the SmartBox workflow is tied to the quality of the initial analog record; any procedural errors during the physical facebow application or earpiece positioning will be mirrored in the subsequent virtual mounting. Any inaccuracies inherent in the mechanical facebow procedure or manual errors during the clinical phase, such as improper earpiece seating or bite fork instability, were replicated in the virtual environment. While the SmartBox ensures a high‐fidelity bridge to the digital workflow, it does not correct for errors made during the physical mounting process; it only ensures that the recorded relationship is precisely preserved during the transition. Future research should include clinical trials to assess the efficacy of the customized tool in real patient contexts. The design and workflow of the SmartBox transfer tool have been submitted for patent registration to the Department of Intellectual Property of Thailand.

## 5. Conclusions

Within the limitations of this study, it can be concluded that the method for transferring the relationship between the maxillary occlusal plane and condylar path from the mechanical to the virtual articulator by using the customized transfer tool is accurate, with no statistically significant differences.

## Author Contributions

Chaimongkon Peampring contributed to conception, formal analysis, funding acquisition, methodology, resources, supervision, validation, review, and editing of the manuscript. Leena Kiratiwudh contributed to conception, data curation, investigation, methodology, and writing the original draft of the manuscript.

## Acknowledgments

This research was technically supported by Thailand Dental Lab.

## Funding

This study was supported by the Research Center of Excellence for Oral Health, Faculty of Dentistry, Prince of Songkla University.

## Disclosure

All authors have read and approved the final version of the manuscript. Chaimongkon Peampring/Leena Kiratiwudh had full access to all of the data in this study and take complete responsibility for the integrity of the data and the accuracy of the data analysis.

## Conflicts of Interest

The authors declare no conflicts of interest.

## Data Availability

The data that support the findings of this study are available upon request from the corresponding author.
